# Highly Active Copolymerization of Ethylene and *N*-Acetyl-*O*-(ω-Alkenyl)-l-Tyrosine Ethyl Esters Catalyzed by Titanium Complex

**DOI:** 10.3390/polym8030064

**Published:** 2016-03-10

**Authors:** Jing Wang, Hongming Li, Runcong Zhang, Xianghui Shi, Jianjun Yi, Jian Wang, Qigu Huang, Wantai Yang

**Affiliations:** 1 State Key Laboratory of Chemical Resource Engineering, Key Laboratory of Carbon Fiber and Functional Polymers, Ministry of Education, the College of Material Science and Engineering, Beijing University of Chemical Technology, Beijing 100029, China; wangjing9917@126.com (J.W.); lihongming010@petrochina.com.cn (H.L.); xzhangrc@163.com (R.Z.); sxhedu1990@163.com (X.S.); yangwt@mail.buct.edu.cn (W.Y.); 2 Lab for Synthetic Resin Research Institution of Petrochemical Technology, China National Petroleum Corporation, Beijing 100083, China; yijianjun@petrochina.com.cn; 3 Liaoyang Petrochemical Corporation, China National Petroleum Corporation, Liaoyang 111003, China; Wangjal@petrochina.com.cn

**Keywords:** *N*-acetyl-*O*-(ω-alkenyl)-l-tyrosine ethyl esters, titanium complex, copolymerization, hydrophilicity

## Abstract

A series of *N*-acetyl-*O*-(ω-alkenyl)-l-tyrosine ethyl esters were synthesized by the reaction of vinyl bromides (4-bromo-1-butene, 6-bromo-1-hexene, 8-bromo-1-octene and 10-bromo-1-decene) with *N*-acetyl-l-tyrosine ethyl ester. ^1^H NMR, elemental analysis, FT-IR, and mass spectra were performed for these *N*-acetyl-*O*-(ω-alkenyl)-l-tyrosine ethyl esters. The novel titanium complex can catalyze the copolymerization of ethylene and *N*-acetyl-*O*-(ω-alkenyl)-l-tyrosine ethyl esters efficiently and the highest catalytic activity was up to 6.86 × 10^4^ gP·(molTi)^−1^·h^−1^. The structures and properties of the obtained copolymers were characterized by FT-IR, (^1^H)^13^C NMR, GPC, DSC, and water contact angle. The results indicated that the obtained copolymers had a uniformly high average molecular weight of 2.85 × 10^5^ g·mol^−1^ and a high incorporation ratio of *N*-acetyl-*O*-(but-3-enyl)-l-tyrosine ethyl ester of 2.65 mol % within the copolymer chain. The units of the comonomer were isolated within the copolymer chains. The insertion of the polar comonomer into a copolymer chain can effectively improve the hydrophilicity of a copolymer.

## 1. Introduction

Ethylene, which has large production, is mostly used to produce non-polar polyethylene in the industrial field. Because polyethylene has excellent performance, uniting chemical and physical properties along with good process ability and low cost, it can be used as an ideal biological material for heart valves, artificial joints, *etc.* after treatment of its surface. However, polyethylene has no polar groups in its polymer chains, resulting in poor compatibility with other materials and also limiting its applications. Recently, the synthesis of new polyolefins with specified functionality or high performance has attracted much attention.

Marks [1] adoptedCGCTiMe_2_/Ph_3_C^+^B(C_6_F_5_)_4_^−^ to catalyze the copolymerization of ethylene and alkenylsilane and obtained functional polyolefin. Sun [2] obtained copolymers of ethylene and11acid vinyl ester via imino-indolate half-titanocene chlorides after activation by MAO. The comonomer incorporation ratio was 1.02 mol % and the catalytic activity was 3.5 × 10^3^ gP/(molTi·h). These investigations showed that metallocene catalysts can catalyze the copolymerization of ethylene and polar monomers. However, the heteroatom from the polar comonomer tends to form a complex with the center metal, resulting in the deactivation of the transition metal catalyst and a decrease in the catalytic activity for polymerization. Non-metallocene catalysts have “tolerance” to heteroatoms, so the synthesis of non-metallocene catalysts has been given much attention. Hu [3] obtained a high molecular weight of functionalized polyolefins containing high contents of polar groups through performing copolymerizations of ethylene and polar comonomers via a bis(phenoxyketimine) group IV early transition metal complexes. Marks [4] investigated whether the bimetallic FI^2^–Ni_2_ catalysts can enhance polar comonomer enchainment selectivity in copolymerization of ethylene and polar-functionalized norbornenes, methylacrylate, or methyl methacrylate, *etc.* The results showed that the comonomer incorporation ratio was more than 10%; however, the catalytic activity was as low as 10^3^ gPE (molNi)^−^^1^·h^−^^1^ and the number-averaged molecular weight of the obtained polymers was only in the thousands. Copolymerization of ethylene with methyl acrylate and other polar monomers was catalyzed by a new palladium complex bearing phosphine phosphonic amide ligands [5]. Guan [6] and Ye [7] synthesized copolymers of ethylene and acrylic monomers with comonomer incorporations of 20 and 3.6 mol %, respectively. Li [8] performed the copolymerization of ethylene and methyl methacrylate with neutral nickel(II) complexes, producing high molecular weight functionalized polyethylene with up to 16.7 mol % of methacrylate units built into the moderately branched polyethylene backbone. Pugh [9] adopted [P, O] palladium complexes to catalyze the copolymerization of ethylene and methyl acrylate with comonomer incorporation of 10 mol %. Nozaki [10,11] prepared copolymers of ethylene and acrylonitrile, allyl halides, allylalcohol, allylamines, allyl acetate, *etc.* using [P-SO_3_] palladium complexes. Jordan [12] studied the copolymerization of ethylene and vinyl fluoride by [P, O]PdMe(py)catalysts. Copolymerizations of ethylene with *N*-vinyl-2-pyrrolidinone and *N*-isopropylacrylamide were performed by [P, O] palladium complexes [13]. In our group’s previous work, copolymers of ethylene and α-olefins or polar monomer were prepared via non-metallocene catalysts with [N, N, O] [14], [N, N, N] [15], and [N, N, O, O] [16,17] ligands. In these investigations, the comonomers used were vinyl organics; biomass based monomers were not involved.

Merrifield set up a method to produce protein by solid synthesis. He fixed amino acids on insoluble resins and then condensed amino acids to the resins in order. Insoluble resin carriers are mainly polystyrene-divinyl benzene crosslinked resin, polyacrylamide, poly-ethylene glycol resin, and their derivatives. However, amino acids cannot be attached on a carrier without introducing enough reactive groups on the carrier. Synthesis of polymers with pendent bioactive molecules without a carrier had attracted much attention in previous years [18]. So far, metathesis (ROMP and ADMET) [19,20,21,22] and atom transfer radical (ATRP) [23] polymerizations, for instance, are extensively used to produce biofunctionalized macromolecules. A unique control of molecular weight, polydispersity, and stereoregularity is offered, however, by metal-catalyzed insertion polymerization. Rieger [24] performed coordination polymerization of carbon monoxide and α-olefins substituted with protected tyrosine or with dipeptide sequences such as tyrosine-glycine, tyrosine-alanine, and tyrosinevaline by palladium. Recently, Jewett and colleagues reported a simple temperature-responsive protein–polymer bioconjugate [25]. They use damber suppression to site-specifically incorporate the noncanonicalazide-functional amino acid *p*-azidophenylalanine(pAzF) into sfGFP at different positions by “click” chemistry.

Herein, we hope to synthesize polymers with pendent bioactive molecules through coordination polymerization. A series of non-metallocene catalysts were synthesized and discussed in terms of their catalytic behavior for the copolymerization of ethylene and *N*-acetyl-*O*-(ω-alkenyl)-l-tyrosine ethyl ester in detail in our previous study [26,27]. Titanium complex exhibited higher catalytic activity for the copolymerization of ethylene and *N*-acetyl-*O*-(ω-alkenyl)-l-tyrosine ethyl ester. In this study, we synthesized polyolefins containing some amino acid groups via catalyzing the copolymerization of ethylene and *N*-acetyl-*O*-(ω-alkenyl)-l-tyrosine ethyl esters by titanium complex/MAO catalyst system.

## 2. Experimental

### 2.1. General Remarks

Aniline (99.8%), *N*-acetyl-l-tyrosine ethyl ester (99%), 4-bromo-1-butene (97%), 6-bromo-1-hexene (97%), 8-bromo-1-octene (97%),10-bromo-1-decene (97%), 2,4,6-trimethylaniline (99%), dichlorodimethylsilane (99.8%), 2,4,6-trifluoroaniline (97%), chlorodiisopropylphosphine (99%), methylmagnesium chloride (CH_3_MgCl) with 22 wt % in THF, and MAO with 10 wt % in toluene were purchased from J&K (Beijing, China). Acetone and diethyl ether were dried by activated Davison 5 Å molecular sieves before use. Toluene and *n*-hexane were purified by refluxing over sodium benzophenone for 48 h before use.

### 2.2. Characterization

^1^H (^13^C) NMR spectra of the ligands and the complexes were recorded on a Varian INOVA 600 MHz spectrometer in deuterated chloroform (CDCl_3_) or deuterateddimethyl sulfoxide (DMSO) solution at 25 °C and tetramethylsilane (TMS) was used as reference. FT-IR spectra were recorded on a Nicolet 5DXC FT-IR spectrograph. The spectra were obtained at 4 cm^−1^ resolution, and average data were obtained from at least 32 scans in the standard wavenumber range from 500 to 4000 cm^−1^. (Nicolet, Madison, AL, America). Mass spectra were recorded by Esquire-LC mass spectroscopy (Bruker, Karlsruhe, Germany), acetone as solvent. Elemental analysis were performed on a PerkinElmer 2400 microanalyzer (Toolso, Taunton, MA, USA), using the combustion method with quantitative oxygen and a thermal conductivity detector. ^1^H (^13^C) NMR spectra of copolymers were performed on a Varian Unity 400 MHz spectrometer (Bruker, Karlsruhe, Germany) at 125 °C, with ortho-dichlorobezene (*d*_4_) as the solvent and tetramethylsilane as reference. The molecular weight (*M*_n_, *M*_w_) and molecular weight distribution (MWD) were measured with a PL-GPC 200 instrument (Varian, Palo Alto, CA, USA), using standard polyethylene (PE) as reference and 1,2,4-trichlorobenzene as solvent at 150 °C. Differential scanning calorimetry(DSC) thermograms were recorded with a PA5000-DSC instrument (Perkin-Elmer, Munich, Germany) at a rate of 10 °C·min^−1^ and determined in the second scan.

### 2.3. Synthesis of N-Acetyl-O-(ω-Alkenyl)-l-Tyrosine Ethyl Ester

*N*-acetyl-*O*-(ω-alkenyl)-l-tyrosine ethyl ester was synthesized according to the literature [24]. A white solid powder (*N*-acetyl-*O*-(but-3-enyl)-l-tyrosine ethyl ester) was obtained with a yield of 38.2%.^1^H NMR (600 MHz, CDCl_3_): δ 7.00 (d, 2H, benzene), δ 6.82(d, 2H, benzene), δ 5.88 (m,1H, C**H**=CH_2_), δ 5.82 (d, 1H, N**H**–CH), δ 5.15–5.25 (m, 2H, –CH_2_–CH=C**H**_2_), δ 4.80 (d, 1H, C**H**–NH), δ 4.25 (m, 2H, C**H**_2_–CH_3_), δ 4.00 (s, 2H, C**H**_2_–O), δ 3.06 (t, 2H, C**H**_2_–CH), δ 2.51 (m, 2H, C**H**_2_–CH), δ 1.99 (s, 3H, C**H**_3_–C=O), δ 1.25 (t, 3H, C**H**_3_–CH_2_); FT-IR (cm^−1^, KBr): 3323 (N–H), 1730 (C=O), 1550 (N–H), 1256 (C–N). Anal. Calc. (%) for C_17_H_23_NO_4_ (305.4 g/mol): C, 69.97; H, 9.05; N, 3.71; found: C, 70.01; H, 9.04; N, 3.68. ESI-MS *m*/*z* calculated for [M+H]^+^. C_17_H_24_NO_4_: 306.40 found 306.46.

*N*-acetyl-*O*-(hex-5-enyl)-l-tyrosine ethyl ester was obtained with a yield of 45.0%. ^1^H NMR (600 MHz, CDCl_3_): δ 6.98 (d, 2H, benzene), δ 6.81 (d, 2H, benzene), δ 5.91(m, 1H, C**H**=CH_2_), δ 5.82 (d, 1H, N**H**–CH), δ 4.96–5.00 (m, 2H, –CH_2_–CH=C**H**_2_), δ 4.82 (d, 1H, C**H**–NH), δ 4.18 (m, 2H, C**H**_2_–CH_3_), δ 3.93 (s, 2H, C**H**_2_–O), δ 3.06 (t, 2H, C**H**_2_=CH), δ 2.13 (m, 2H, C**H**_2_–CH), δ 1.99 (s, 3H, C**H**_3_–C), δ 1.79 (m, 2H, C**H**_2_–CH_2_), δ 1.56 (m, 2H, C**H**_2_–CH_2_), δ 1.26 (t, 3H, C**H**_3_–CH_2_); FT-IR (cm^−1^, KBr): 3365 (N–H), 1722 (C=O), 1513 (N–H), 1268 (C–N). Anal. Calc. (%) for C_19_H_27_NO_4_ (333.20 g/mol): C, 68.47; H, 7.51; N, 4.20; found: C, 68.48; H, 7.59; N, 4.25. ESI-MS *m*/*z* calculated for [M+H]^+^. C_19_H_28_NO_4_: 334.20 found 334.31.

*N*-acetyl-*O*-(oct-7-enyl)-l-tyrosine ethyl ester was obtained with a yield of 52.9%, ^1^H NMR (600 MHz, CDCl_3_): δ 6.99 (d, 2H, benzene), δ 6.82 (d, 2H, benzene), δ 5.91 (m, 1H, C**H**=CH_2_), δ 5.82 (d, 1H, N**H**–**C**H), δ 4.94–5.03 (m, 2H, –CH_2_–CH=C**H**_2_), δ 4.80 (d, 1H, C**H**–NH), δ 4.17 (m, 2H, C**H**_2_–CH_3_), δ 3.91 (s, 2H, C**H**_2_–O), δ 3.05 (t, 2H, C**H**_2_=CH), δ 2.06 (m, 2H, C**H**_2_–CH), δ 1.97 (s, 3H, C**H**_3_–C), δ 1.3–1.5 (m, 8H, O–C**H**_2_–C**H_2_**–C**H_2_**–C**H_2_**),δ 1.26 (t, 3H, C**H**_3_–CH_2_); FT-IR (cm^−1^, KBr): 3311 (N–H), 1751 (C=O), 1545 (N–H), 1266 (C–N). Anal. Calc. (%) for C_21_H_31_NO_4_ (361.32 g/mol): C, 69.77; H, 8.60; N, 3.88; found: C, 69.78; H, 8.57; N, 3.85.ESI-MS *m*/*z* calculated for [M+H]^+^. C_21_H_32_NO_4_: 362.32 found 362.41.

*N*-acetyl-*O*-(dec-9-enyl)-l-tyrosine ethyl ester was obtained with a yield of 45.2%. ^1^H NMR (600 MHz, CDCl_3_): δ 6.99 (d, 2H, benzene), δ 6.82 (d, 2H, benzene), δ 5.89 (m, 1H, C**H**=CH_2_), δ 5.81 (d, 1H, N**H**–**C**H), δ 4.94–4.99 (m, 2H, –CH_2_–CH=C**H**_2_), δ 4.82 (d, 1H, C**H**–NH), δ 4.19 (m, 2H, C**H**_2_–CH_3_), δ 3.94 (s, 3H, C**H**_2_–O), δ 3.07 (t, 2H, C**H**_2_=CH), δ 2.05 (m, 2H, C**H**_2_–CH), δ 2.00 (s, 3H, C**H**_3_–C), δ 1.42–1.34 (m, 10H, C**H**_2_–CH_2_), δ 1.26 (t, 3H, C**H**_3_–CH_2_); FT-IR (cm^−1^,KBr): 3324 (N–H), 1728 (C=O), 1552 (N–H), 1259 (C–N). Anal. Calc. (%) for C_23_H_35_NO_4_ (389.3 g/mol): C, 70.92; H, 8.97; N, 3.61; found: C, 70.90; H, 9.00; N, 3.59. ESI-MS *m*/*z* calculated for [M+H]^+^. C_19_H_26_NO_4_: 390.30 found 390.36.

### 2.4. Synthesis of Catalyst Precursors

**Figure 1:**
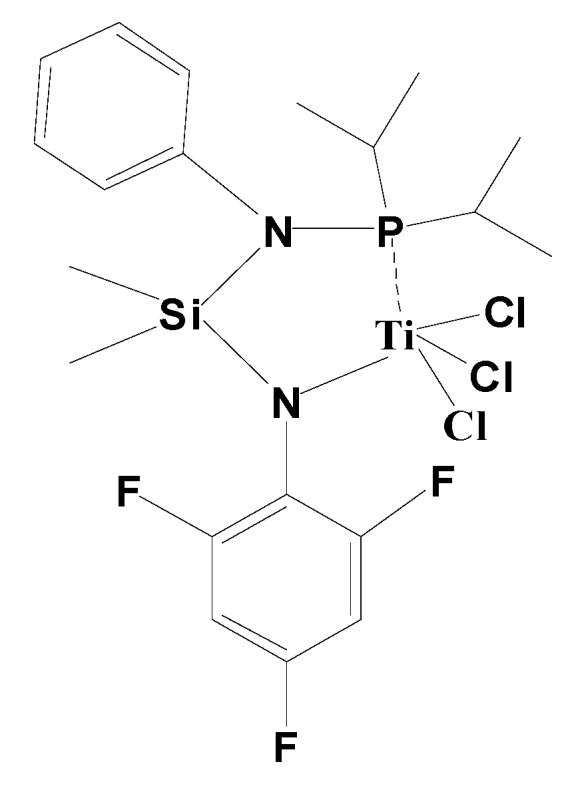
Structure of titanium complex.

Catalyst precursors (Figure 1) were prepared according to the literature [24].

### 2.5. Polymerization Procedure

Polymerization were carried out in a 300 mL Schlenk-type glassware with a magnetic stirrer. Freshly distilled toluene (80 mL), desired amount of catalyst and MAO were added into the reactor. After 15 min for preactivation, *N*-acetyl-*O*-(ω-alkenyl)-l-tyrosine ethyl esters was injected into the polymerization system. The comonomers were treated by an equivalent of AlEt_3_ as a protect reagent prior to use. The polymerization carried out for 10 minutes. The reaction was terminated with 10 wt % HCl in alcohol. The obtained product was filtered, washed with distilled water, and then dried at 80 °C *in vacuo* to constant weight.

## 3. Results and Discussion

**Scheme 1:**
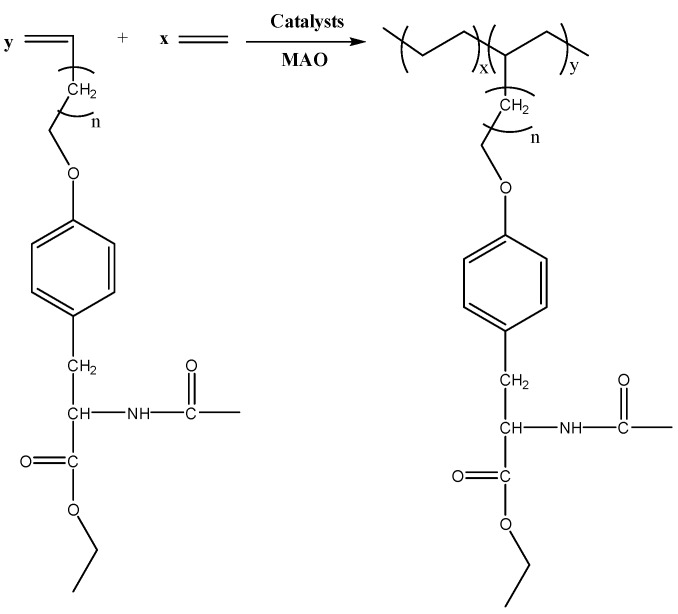
Copolymerization of ethylene and *N*-acetyl-*O*-(ω-alkenyl)-l-tyrosine ethyl esters. *n* = 1, 3, 5, 7.

Copolymerizations of ethylene and different *N*-acetyl-*O*-(ω-alkenyl)-l-tyrosine ethyl esters were performed using a titanium complex/MAO catalyst system (Scheme 1).

**Figure 2:**
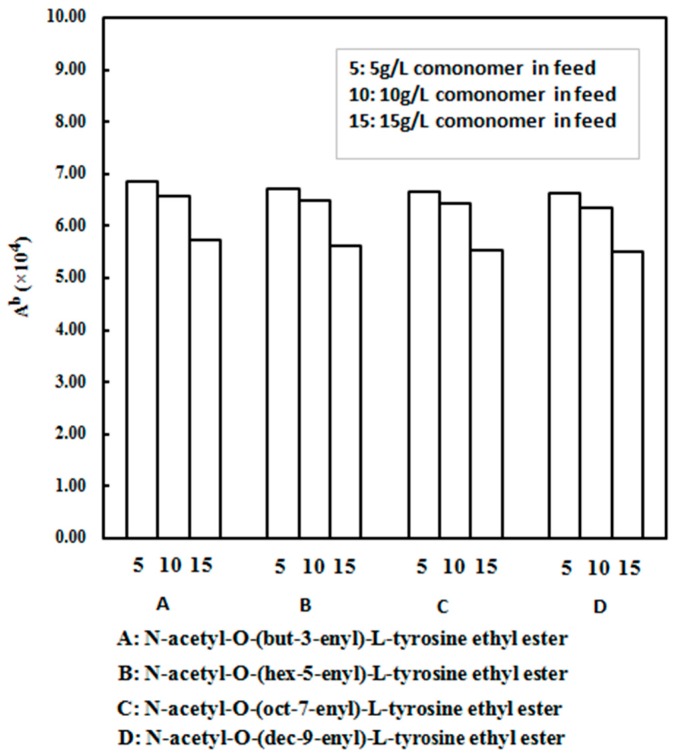
Activity diagrams for the copolymerization of ethylene and different comonomers with titanium complex.

As can be seen in Figure 2, copolymerization of ethylene and four kinds of *N*-acetyl-*O*-(ω-alkenyl)-l-tyrosine ethyl esters with different carbon chain lengths were performed with a titanium complex/MAO catalyst system. The catalytic activity was similar for the copolymerization of ethylene and different comonomer, and the comonomer incorporation ratio was similar, too. With an increase in the comonomer concentration, the catalytic activity decreased. It is possible that an excess of comonomer deactivated the active center. It is not obvious, but the catalytic activity for copolymerization of ethylene and *N*-acetyl-*O*-(ω-alkenyl)-l-tyrosine ethyl ester was the highest among those copolymerizations of ethylene and different *N*-acetyl-*O*-(ω-alkenyl)-l-tyrosine ethyl esters catalyzed by the titanium complex/MAO catalyst system (Run 2, 3, 4 in Table 1). It is possible that the “poison” of amino acid ester to the active centers is hindered because of an aromatic ring preventing the polar group from approaching the active centers.

**Figure 3:**
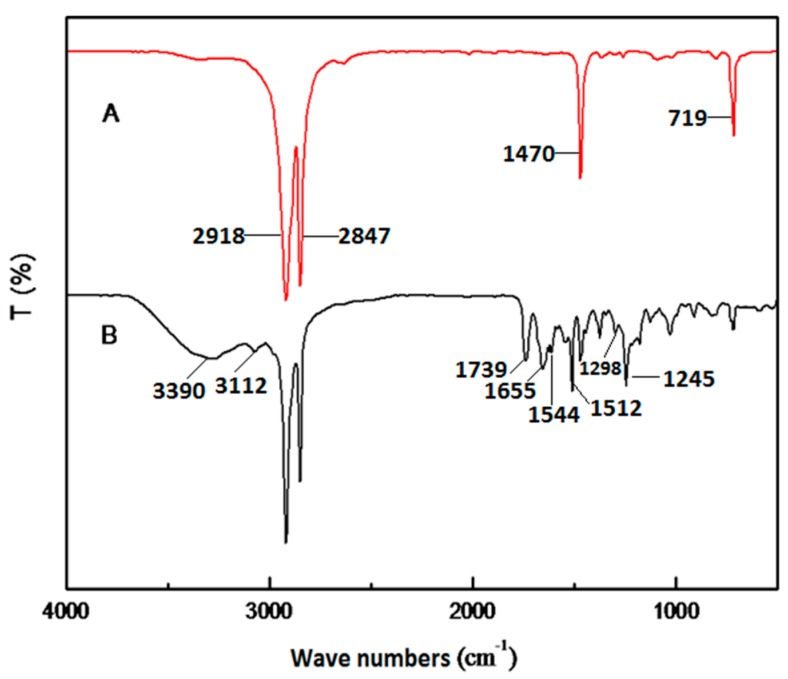
FT-IR spectra of ethylene homopolymer (**A**, run 1 in Table 1) and ethylene/*N*-acetyl-*O*-(but-3-enyl)-l-tyrosine ethyl ester copolymer (**B**, run 3 in Table 1).

The FT-IR spectra of the obtained polymers showed a band at 1739 cm^−1^ (Figure 3B), which is attributed to C=O vibration absorption of the saturated ester from the branched group. Bands at 2918, 2847, 1470, and 719 cm^−1^were characters of linear polyethylene (Figure 3). Fan reported that C=O vibration absorption of *N*-acetyl-*O*-(ω-alkenyl)-l-tyrosine ethyl ester was at 1760 cm^−1^ [28]; however, herein, the bands of C=O of the copolymer B moved toward the red area and became 1739 cm^−1^ [29]. In addition, bands at 3390 and 3112 cm^−1^ were attributed to N–H vibration absorption, indicating that molecular association took place. Bands at 1655, 1544, 1512, and 1298 cm^−1^ were attributed to stretching vibration absorption and bending vibration absorption of C=O, N–H, and C–N. Bending vibration absorptions of C–O on the ester group were shown at 1245 cm^−1^. The results indicated that the comonomer *N*-acetyl-*O*-(but-3-enyl)-l-tyrosine ethyl ester had been incorporated into the copolymer chains.

**Figure 4:**
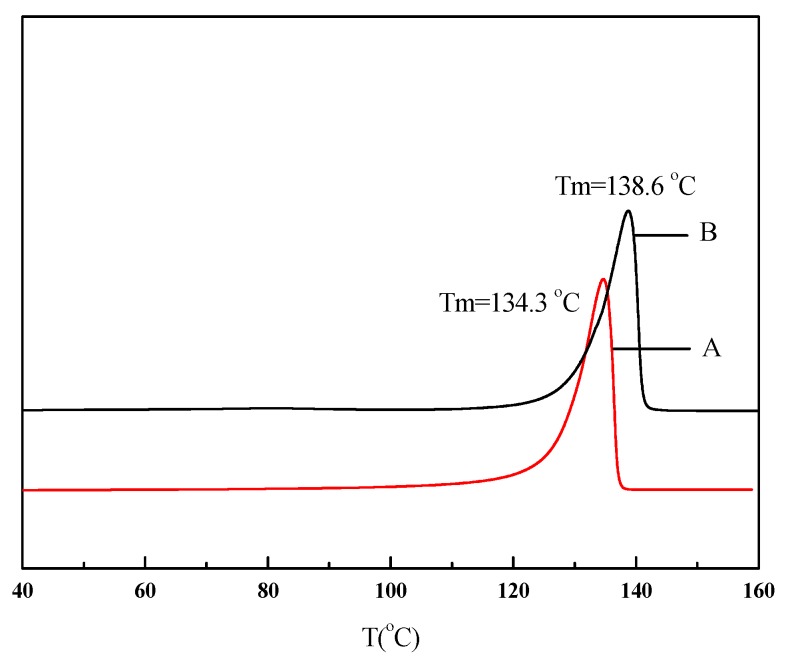
DSC spectra (the second heating) of ethylene homopolymer (**A**, run 1 in Table 1) and ethylene/*N*-acetyl-*O*-(but-3-enyl)-l-tyrosine ethyl ester copolymers (**B**, run 3 in Table 1).

As shown in Figure 4, the melting point of polyethylene (Figure 4A) and the copolymer (Figure 4B) were 134.3 and 138.6 °C, respectively. The molecular weight of copolymer was lower than that of polyethylene, but the melting point of the copolymer was higher than that of polyethylene, which can be attributed to the hydrogen bonding interaction between the side groups confirmed by the FT-IR spectrum (Figure 3). The melting enthalpy and crystallinity of polyethylene were 197.7 J/g and 68.4%, respectively. The melting enthalpy of the copolymer was 147.5 J/g; its crystallinity was 51.2%, obviously lower than that of polyethylene. These results implied that the insertion of *N*-acetyl-*O*-(but-3-enyl)-l-tyrosine ethyl ester destroyed the regularity of the polymer chain, resulting in the reduction of crystallinity.

**Figure 5:**
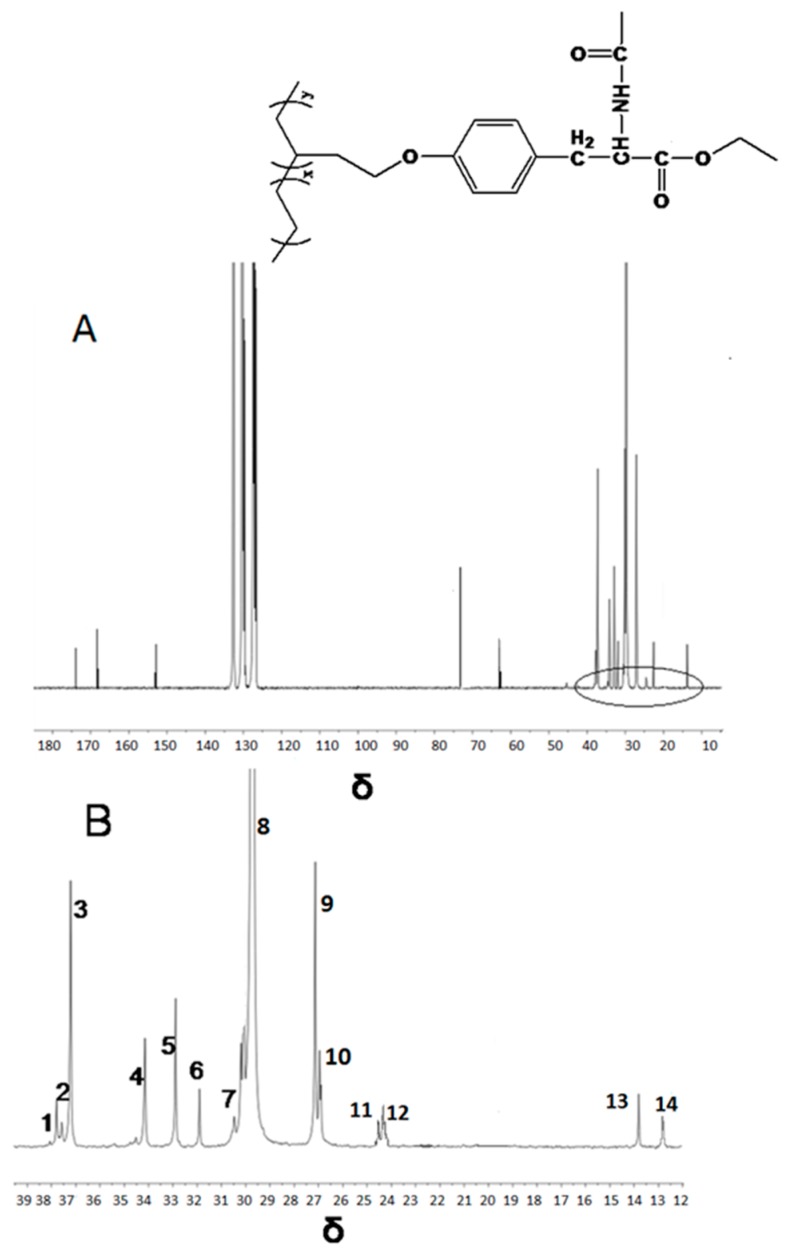
High-temperature solution^13^C NMR spectra (**A**); and expandedview (δ = 10–40 ppm) (**B**) of the copolymer of ethylene/*N*-acetyl-*O*-(but-3-enyl)-l-tyrosine ethyl ester with comonomer insertion ratio 2.24 mol % from run 3 in Table 1.

A high temperature solution ^13^C NMR spectrum of the copolymer of ethylene and *N*-acetyl-*O*-(but-3-enyl)-l-tyrosine ethyl ester (run 3 in Table 1) is shown in Figure 5A. Signals at δ = 153.67, 132.26, 127.39, and 126.13 ppm represent the carbon atoms on the benzene ring in tyrosine ethyl ester. The signal at δ = 174.07 ppm is attributed to the carbon atom from the amido group and the signal at δ = 168.62 ppm is attributed to the carbon from the ester group. Methylene carbon attached to the phenoxy group showed at δ = 73.03 ppm, methylene carbon of ethyl ester is observed at δ = 63.07 ppm, and the α-carbon of tyrosine ethyl ester is observed at δ = 62.98 ppm. Carbons from benzyl methylene showed at δ = 37.20 ppm, carbons from methine on the main chain showed at δ = 33.01 ppm, and methylene groups that connect the main chain and Tyr showed at δ = 30.30 ppm. Carbons from acetyl methyl and ester methyl showed at δ = 13.85 or 12.63 ppm, respectively. These results indicated that the comonomer was inserted into the polyethylene chain.

An expanded view (δ = 10–40 ppm) of ^13^C NMR spectrum is shown in Figure 5B. Carbon atoms between 10 and 40 ppm were assigned in Table 2. The Randall method [30] was used to calculate the content of different sequence structures, and then the comonomer insertion ratio as well as the reactivity ratio and the average sequence length of the two monomers was calculated. The calculation results showed that the reactivity ratios of ethylene and *N*-acetyl-*O*-(but-3-enyl)-l-tyrosine ethyl ester were 149.31 and 0.083, respectively. Moreover, no continuous segment of *N*-acetyl-*O*-(but-3-enyl)-l-tyrosine ethyl ester was found within the copolymer chain ([BBB] = 0) (Table 3). So we can infer that the units of comonomer were isolated within the copolymer chains, which can give the copolymers good potential applications, such as drug vector, support for synthetic protein, and so on.

The chain transfer in the copolymerization process catalyzed by the titanium complex has two main patterns: β-H transfers to active center and monomer, and growing chain transfer to MAO. A polymer obtained from the former has an unsaturated end group, while a polymer obtained from the latter has a saturated end group. Chain termination reaction of olefin polymerization catalyzed by the titanium complex can also obtain a polymer with a saturated end group. From Figure 5B, there is no signal of the unsaturated carbon–carbon double bond, but a signal at δ = 13–14 ppm assigned to methyl was observed, implying that the obtained copolymer of ethylene and *N*-acetyl-*O*-(but-3-enyl)-l-tyrosine ethyl ester was featured with methyl at the chain end, which confirmed that the chain transfer to MAO was the dominant fashion.

**Figure 6:**
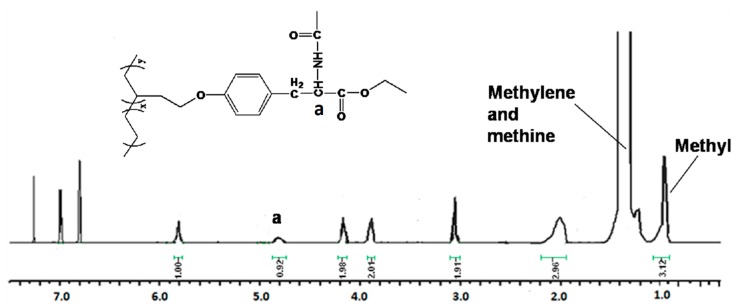
^1^H NMR spectrum of the copolymer of ethylene/*N*-acetyl-*O*-(but-3-enyl)-l-tyrosine ethyl ester from run 3 in Table 2.

The incorporations of *N*-acetyl-*O*-(but-3-enyl)-l-tyrosine ethyl ester into the copolymers have been characterized by ^1^H NMR (Figure 6). The existence of a comonomer can be determined by the signal of methine protons of CH–N at 4.8 ppm. The incorporation ratio of *N*-acetyl-*O*-(but-3-enyl)-l-tyrosine ethyl ester into the copolymer chains determined by the integrated intensity ratio of signal at 4.8 ppm to the signals at 1.2–1.4 ppm.

**Figure 7:**
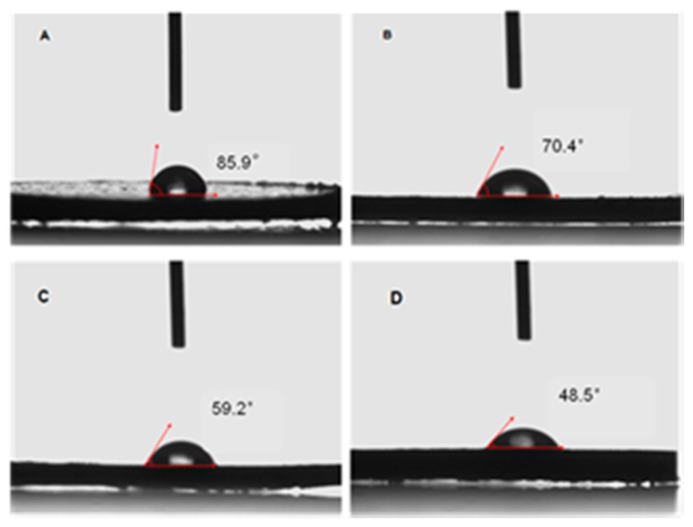
Water contact angle of polyethylene (**A**, PE) from run 1 in Table 1 ethylene/*N*-acetyl-*O*-(but-3-enyl)-l-tyrosine ethyl ester copolymers; (**B**) comonomer insertion ratio of 1.16 mol % from run 2 in Table 1; (**C**) comonomer insertion ratio of 2.24 mol % from run 3 in Table 1; (**D**) comonomer insertion ratio of 2.65 mol % from run 4 in Table 1).

The water contact angle of polyethylene and ethylene/*N*-acetyl-*O*-(but-3-enyl)-l-tyrosine ethyl ester copolymers was measured (Figure 7). Generally speaking, the water contact angle will be smaller when the polarity of the sample surface becomes stronger. The water contact angle of the polyethylene film was 85.9°, as shown in Figure 7A; the water contact angle of the films of the copolymers of ethylene/*N*-acetyl-*O*-(but-3-enyl)-l-tyrosine ethyl ester were 70.4°, 59.2°, and 48.5° when the comonomer insertion ratio were 1.16, 2.24 and 2.65 mol %, respectively (Figure 7B–D). With the comonomer insertion ratio increasing, the water contact angle on the sample surface became smaller—that is to say, the hydrophilicity of the obtained copolymers became stronger as the comonomer insertion ratio increased. From the above results, we can infer that insertion of the polar comonomer into a copolymer chain can effectively improve the hydrophilicity of the copolymer.

## 4. Conclusions

The copolymers of ethylene and *N*-acetyl-*O*-(ω-alkenyl)-l-tyrosine ethyl esters with hydrophilic property were efficiently prepared by titanium complex after activation by MAO, with high catalytic activity of 6.86 × 10^4^ gP·(molTi)^−1^·h^−1^. The insertion ratio was as high as 2.65 mol %. ^13^C NMR results revealed that the units of the comonomer were isolated within the copolymer chains. Copolymers exhibited higher melting temperature than the polyethylene determined by DSC. MWDs of the obtained copolymers were smaller than 3, broader than these of polyethylene. Furthermore, insertion of the comonomer into the copolymer chains can effectively improve the hydrophilicity of the copolymer.

## Figures and Tables

**Table 1:** Copolymerization of ethylene and *N*-acetyl-*O*-(ω-alkenyl)-l-tyrosine ethyl esters with titanium complex.

Run	Comonomer	Comon ^a^ (g/L)	A ^b^ (×10^4^)	*T*_m_^c^ (°C)	N-Cont ^d^ (mol%)	*M*_w_^e^ (×10^5^)	*M*_w_/*M*_n_^e^
1	None	0	170	134.3	0	8.27	1.77
2	*N*-acetyl-*O*-(but-3-enyl)-l-tyrosine ethyl ester	5	6.86	137.9	1.16	2.75	2.88
3	*N*-acetyl-*O*-(but-3-enyl)-l-tyrosine ethyl ester	10	6.57	138.4	2.24	2.76	2.87
4	*N*-acetyl-*O*-(but-3-enyl)-l-tyrosine ethyl ester	15	5.73	139.0	2.65	2.85	2.91
5	*N*-acetyl-*O*-(hex-5-enyl)-l-tyrosine ethyl ester	5	6.71	138.0	1.13	2.72	2.85
6	*N*-acetyl-*O*-(hex-5-enyl)-l-tyrosine ethyl ester	10	6.49	138.6	2.19	2.74	2.86
7	*N*-acetyl-*O*-(hex-5-enyl)-l-tyrosine ethyl ester	15	5.61	139.3	2.60	2.87	2.89
8	*N*-acetyl-*O*-(oct-7-enyl)-l-tyrosine ethyl ester	5	6.67	137.8	1.12	2.76	2.87
9	*N*-acetyl-*O*-(oct-7-enyl)-l-tyrosine ethyl ester	10	6.43	138.5	2.21	2.79	2.85
10	*N*-acetyl-*O*-(oct-7-enyl)-l-tyrosine ethyl ester	15	5.53	139.2	2.59	2.89	2.90
11	*N*-acetyl-*O*-(dec-9-enyl)-l-tyrosine ethyl ester	5	6.63	137.7	1.11	2.73	2.86
12	*N*-acetyl-*O*-(dec-9-enyl)-l-tyrosine ethyl ester	10	6.34	138.4	2.28	2.70	2.79
13	*N*-acetyl-*O*-(dec-9-enyl)-l-tyrosine ethyl ester	15	5.50	139.1	2.57	2.82	2.88

^a^ comonomer; ^b^ activity of catalysts, g PE(molTi)^−1^·h^−1^; ^c^ results of DSC; ^d^ incorporation content of comonomer, results of ^1^H NMR. ^e^ results of GPC. Reaction conditions: pressure of ethylene, 0.2 × 10^6^ Pa; catalyst concentration, 2.0 × 10^−4^ mol·L^−1^; Al/M in mol, 600; reaction temperature, 60 °C; dissolvent, toluene, 100 mL; polymerization time, 10 min.

**Table 2:** ^13^C NMR data of carbon atoms on the copolymer of ethylene and *N*-acetyl-*O*-(but-3-enyl)-l-tyrosine ethyl ester.

Peak NO.	Carbon Assignment	Sequence Assignment	Chemical Shift	
			Calculated	Found
1	αα	EBBE	38.03	37.91
2	Methine	EBBE	37.69	37.66
3	Methine	EBB+BBE	37.01	37.15
4	αδ^+^	BBEE+EEBB	34.01	34.12
5	αδ^+^	EBEE+EEBE	32.91	32.87
6	γγ	BEEB	30.92	31.87
7	γδ^+^	BEEE+EEEB	30.47	30.50
8	δ^+^δ^+^	(EEE)n	29.98	29.78
9	βδ^+^	ETEE+EETE	27.27	26.90
10	2B_2_^+^	EBB+BBE	26.80	26.86
11	ββ	EBEBE	24.54	24.50
12	ββ	BBEBE+BBEBB	24.39	24.32
13	Methyl	EBE	13. 80	13.86
14	Methyl	BBE+EBB	12.41	12.60

E: unit of ethylene; B: unit of *N*-acetyl-*O*-(but-3-enyl)-l-tyrosine ethyl ester.

**Table 3:** The content of different sequences for the copolymer and reactivity ratios for the monomers.

Content of Sequences						Mole Fractions		Reactivity Ratio	
[EBE]	[EBB]	[BBB]	[BEB]	[EEB]	[EEE]	[E]	[B]	*r*_E_	*r*_B_
2.163	0.008	0	0.072	4.326	93.431	0.9778	0.0222	149.31	0.083

E: unit of ethylene; B: unit of *N*-acetyl-*O*-(but-3-enyl)-l-tyrosine ethyl ester.
